# A Pilot Study of the Real-World Impact of Digital Cognitive Behavioral Therapy for Inflammatory Bowel Disease (COMPASS-IBD) on Inflammation, Disease Activity, and Healthcare Use

**DOI:** 10.1097/PSY.0000000000001457

**Published:** 2025-11-21

**Authors:** Natasha Seaton, Sophie Harding, Annie S. K. Jones, Tsz Wai Chow, Valeria Mondelli, Joanna L. Hudson, Rona Moss-Morris

**Affiliations:** aff1 Department of Psychology, Institute of Psychiatry, Psychology and Neuroscience, King’s College London, London, UK (Seaton, Harding, Jones, Chow, Hudson, Moss-Morris); aff2 Department of Psychological Medicine, The University of Auckland, Auckland, New Zealand (Jones); aff3 Department of Psychological Medicine, Institute of Psychiatry, Psychology and Neuroscience, King’s College London, London, UK (Mondelli)

**Keywords:** inflammatory bowel disease, CBT, depression, anxiety, inflammation, fecal calprotectin, CRP, healthcare usage, psychoneuroimmunology, symptoms

## Abstract

**Objective:**

Inflammatory bowel disease (IBD) is commonly accompanied by psychological distress, which may worsen disease activity through gut-brain axis mechanisms. Psychological interventions including cognitive behavioral therapy (CBT) seem to reduce distress and inflammation in IBD. However, accessibility to psychological care remains limited. COMPASS-IBD, a digital CBT intervention tailored to IBD, aims to address these gaps.

This nested exploratory, real-world study (NCT05330299) assessed the effectiveness of COMPASS-IBD in reducing inflammation, disease activity, and health care use in IBD patients. In addition, it examined relationships between changes in psychological distress and disease indicators.

**Methods:**

Adults with IBD experiencing psychological distress were recruited from a large gastroenterology service, and enrolled in COMPASS-IBD. Disease-related primary outcomes were faecal calprotectin (FCP), C-reactive protein (CRP), and self-reported disease activity (SRDA). Secondary outcomes included other inflammatory biomarkers (neutrophils, monocytes, lymphocytes, white blood cells (WBC), ferritin, flare frequency, and health care usage. The Patient Health Questionnaire-Anxiety and Depression Scale (PHQ-ADS) measured distress. Mixed-effects models evaluated outcomes at baseline, 12 weeks, and 6 months.

**Results:**

Sixty-five participants were included. There were significant reductions in CRP (*d* range: −0.47 to −0.53, *P*<.01), but not FCP (*d* range: −0.38 to −0.44, *P*<.10) or SRDA (*d*=−0.28, *P*=.08). Significant improvements were observed in WBC and neutrophil counts (*P*<.05), flare frequency (*P*<.01), and psychological distress (*P*<.001). Some types of health care usage were reduced (*P*<.05). Associations between distress and primary outcomes were nonsignificant.

**Conclusions:**

This pilot suggests COMPASS-IBD may reduce inflammation levels, health care use, and psychological distress. However, analyses were underpowered. Larger randomized trials are needed to confirm findings, establish cost-effectiveness, and explore underlying gut-brain mechanisms.

## INTRODUCTION

Inflammatory bowel disease (IBD), comprising Crohn disease (CD) and ulcerative colitis (UC), is a chronic autoimmune inflammatory condition of the gastrointestinal tract. IBD has a relapsing-remitting disease course, with periods of increased disease activity referred to as flares. Symptoms are debilitating and heterogeneous, with reported prevalence of diarrhea at 80%, abdominal pain at 62%, fecal incontinence at 54%, fatigue at 56%, urgency at 40% and rectal bleeding at 22%.^1–4^ Immunomodulatory medications and in some cases liquid diets and surgery are used to treat the condition.^5^ Recent development and increased availability of biologicals has vastly improved IBD care.^6^ Despite this, many patients continue to experience significant disease-related morbidity and functional impairment.^7–9^ Moreover, these pharmacological therapies have negative side effects^10–14^ and high financial costs, which are not offset by reductions in other procedures, such as surgeries.^15,16^

Comorbid mood disorders are common in IBD, with prevalence estimated at 32.1% for anxiety and 25.3% for depression.^17^ Rates increase to 57.6% and 38.9%, respectively, when disease is active.^17^ A diagnosis of depression and/or anxiety increases the risk of subsequent negative health outcomes (eg, hospitalizations, surgery),^18^ even when baseline inflammation is accounted for.^19^ This may be one reason underlying the 55% increased health care costs seen in IBD patients who have comorbid depression or anxiety.^20^ Although anxiety and depression are often considered separately, studies have shown that there is significant overlap in standardized measures of these constructs in people with chronic medical conditions. Composite measures of anxiety and depression, labeled psychological distress, have been validated in these populations.^21,22^ Hence, this term will be used to refer collectively to symptoms of anxiety and depression.

A recent meta-analysis of randomized controlled trials (RCTs) suggested that treating psychological distress improves levels of fecal calprotectin (FCP), C-Reactive Protein (CRP), and general inflammation in IBD.^23^ The meta-analysis showed that larger effects on inflammation were seen for (1) psychological therapies, over exercise and antidepressant interventions, and (2) interventions with larger effect sizes for psychological distress.^23^ Although RCTs are the gold standard for evaluating efficacy, they do not capture how interventions perform under real-world clinical conditions. Naturalistic implementation studies are therefore needed to determine if these effects persist in typical health care settings. Importantly, distress is an independent risk factor for health care usage, after inflammation is controlled,^24–27^ potentially due to influence on symptom perception^28–30^ or help-seeking behavior.^31^ Many existing psychological intervention studies in IBD focus on inflammatory biomarkers without examining health care usage, even though interventions targeting distress may confer benefits across both dimensions.

Despite the promise of psychological therapies within IBD care to (1) treat comorbid psychological distress, (2) confer additional inflammatory benefit with minimal side effects and at low cost (in the UK, a course of face-to-face CBT costs ~£480–£800),^32^ and (3) reduce health care usage,^33,34^ only 2% of UK IBD services adhere to the national standards of having a clinical psychologist (0.5 whole-time-equivalent per 250,000 people).^35,36^ Therefore, developing and implementing effective, low-cost, and scalable psychological interventions remains an unresolved priority in IBD. Digital CBT may help to address this gap, given that effect sizes are comparable with face-to-face therapy, but with greatly reduced therapist time.^37^

However, effect sizes for psychological therapies in IBD and other chronic illness populations are often small,^38,39^ potentially due to insufficient tailoring to the concerns of living with IBD.^40^ Because larger improvements in psychological distress are associated with greater reductions in inflammatory biomarkers,^23^ a more targeted intervention may increase the benefit for both psychological and disease-related outcomes. The COMPASS program is a therapist-supported, digital intervention originally developed to support patients experiencing psychological distress in the context of living with long-term conditions (LTCs), based on the transdiagnostic model of adjustment to LTCs (TMA-LTC).^41^ It consists of 11 online modules that target mechanisms known to sustain psychological distress in LTCs.^42^ The online modules are accompanied by up to six 30-minute appointments with a therapist, through video conferencing or telephone call, aligned with UK Talking Therapy Services.^42–45^ A recent RCT showed that the transdiagnostic version of COMPASS effectively reduced psychological distress compared with standard LTC-charity support, with a large effect size of 0.71.^46^ Almost half the sample had IBD, and qualitative findings indicated that IBD-specific tailoring may enhance relevance,^47^ and thus has potential to increase effect sizes for mood and disease-related outcomes.

Therefore, COMPASS-IBD was developed^47^ and tested in a real-world longitudinal study, where the treatment was implemented into a large secondary care service in London, UK.^48^ The main outcomes paper (reported elsewhere^49^) demonstrated that COMPASS-IBD effectively treated psychological distress between baseline and the post-intervention timepoint. The current study aims to extend those findings to examine whether COMPASS-IBD improves inflammatory disease activity, self-reported clinical activity, and health care usage. This analysis additionally included 6-month data to assess lasting effects beyond a pre-post analysis. This study relied on both self-reported questionnaire data and extraction of inflammatory biomarkers from medical records. By evaluating these outcomes in a naturalistic real-world setting, we sought to determine whether a digital CBT program with therapist support could confer lasting benefits for both inflammation and health care usage in IBD.

### Study aims

The aims of this study were to:

1. Determine the potential effectiveness of COMPASS-IBD, a digital CBT program with therapist support, at improving disease indicators in a real-world setting. Primary outcomes were FCP, CRP, and self-reported disease activity. Secondary outcomes included additional biological and/or health outcomes, such as self-reported disease information (eg, flare frequency and severity), self-reported health care usage, and additional inflammatory biomarkers collected from medical records (levels of white blood cells, ferritin, lymphocytes, neutrophils, and monocytes).
2. Examine whether changes in primary outcomes were associated with changes in psychological distress.

## MATERIALS AND METHODS

### Study Design

This is an exploratory analysis of data from a real-world longitudinal study implementing digital screening and treatment for distress (COMPASS-IBD) for adults with IBD in a UK National Health Service (NHS) secondary care setting. The trial was registered on ClinicalTrials.gov (NCT05330299). The protocol and main outcomes paper are published.^48,49^ In the main study, IBD patients were identified as experiencing clinical distress either through an online digital screening tool (IMPARTS, https://imparts.org/about/) or through clinician referral at Guy’s at St Thomas’s NHS Foundation Trust. Eligible participants were enrolled onto a digital CBT program, tailored to the challenges of living with IBD (COMPASS-IBD). Assessments took place at baseline, post-treatment (12 wk), and at follow-up (6 mo). The study received ethical approval from the Health Research Authority and from North West—Greater Manchester East Research Ethics Committee (22/NW/0224).

### Sample and Recruitment

IBD patients were recruited from November 2022 to September 2023 from a large gastroenterology service, with data collection ceasing in July 2024. Details of recruitment, triage, and participant flow can be found in the protocol and main outcomes papers.^48,49^ In brief, IBD patients were identified as experiencing at least mild psychological distress (≥10 on the Patient Health Questionnaire-Anxiety and Depression Scale (PHQ-ADS)^50^) by completing digital mental health screening before a routine IBD care appointment. Patients could also be highlighted as experiencing distress through direct referrals from a clinician to the IBD psychology service. Potentially eligible patients were contacted by a member of the IBD Psychology service to complete screening for the COMPASS-IBD study. Patients were eligible if they: (i) experienced mild-severe distress, (ii) demonstrated no active/intentional suicidal ideation, (iii) had distress related to their IBD, (iv) had English proficiency, (v) had access to a device to complete the online intervention, and (vi) provided informed consent to participate in the research. Participants were excluded if there was evidence of: (i) substance dependency, cognitive impairment, or a severe mental health condition, (ii) acute suicidal risk (recent serious suicidal intent and/or planning), (iii) current receipt of psychological treatment or on a waitlist to be treated within the next 6 months, and (iv) distress unrelated to IBD.

### Procedure

Eligible participants were sent a participant information sheet (with the option to discuss the study with a member of the research team) and an online consent form to participate in the real-world evaluation of COMPASS IBD. Consent included permitting researchers to view and extract data from medical records and an additional optional consent for the submission of stool samples to assess FCP at baseline and 12 weeks. Upon completion of the consent form, participants were sent an online baseline questionnaire. After completion of the baseline questionnaire, participants were enrolled into the COMPASS-IBD intervention and assigned a therapist. Online questionnaires were sent 12 weeks (post-intervention) and 6 months (follow-up) after baseline. If participants consented to provide a stool sample, they were sent a collection kit with instructions at baseline and post-intervention. If participants had completed a stool sample for their medical team as part of routine care within 3 months of the post-intervention timepoint, this data could be used instead. Relevant medical data were extracted from participants’ electronic hospital records pre-intervention, post-intervention (12 wk) and at follow-up (6 mo). Two researchers (N.S. and T.W.C.) independently extracted data into case report forms. Data were considered acceptable if they were taken within 3 months of the specific study timepoint. Results of extraction were compared, and differences were resolved by discussion.

### Intervention

The COMPASS program, including session content, format, and the role of the therapist, has been detailed elsewhere.^42,46^ Briefly, COMPASS is a personalized, online CBT program completed over 12 weeks. Participants receive six 30-minute appointments with a trained therapist (through video conferencing or phone call, with optional text support). The program uses evidence-based CBT strategies through online modules covering psychoeducation, patient experiences, goal setting, and interactive tasks. These modules address topics such as managing long-term condition uncertainty, unhelpful thoughts, symptoms, and establishing routines. COMPASS-IBD includes the format and core components of COMPASS, while adapting specific content to the challenges of living with IBD.^47^ COMPASS-IBD was supported by trainee clinical psychologists who received COMPASS-specific training and supervision from 2 clinical psychologists: one from the gastroenterology service as part of clinical training (AD), one from an external clinical psychologist with expertise in COMPASS (AW). Users are considered adherent to the program if they attend ≥3 therapist appointments and complete ≥5 online sessions.

### Measures

The measures described below pertain to the exploratory analysis. The primary and secondary outcomes for the main real-world implementation-focused study are described elsewhere.^48,49^

#### Primary Outcomes

The primary outcomes for this study were measures of IBD activity: FCP, CRP, and self-reported disease activity (SRDA).

FCP is a protein, typically presented in neutrophils, that is a very sensitive marker of inflammation in the gastrointestinal tract.^51^ It is used in IBD diagnosis, disease monitoring, informing treatment decisions, and is able to predict relapse in IBD.^52^ Levels <100 ug/g reflect minimal inflammation; ≥100 and <250 ug/g represents possible inflammation and ≥250 ug/g indicates active inflammation.^53^

CRP is a serum biomarker produced by the liver in response to acute inflammation. Although it is systemic, it is well-established as a biomarker for monitoring IBD activity given its significant association with endoscopic disease activity.^54,55^ Guidelines for interpreting CRP stipulate healthy levels at <0.3 mg/dL and acceptable levels ranging 0.3–1.0 mg/dL. Values between 1.0 and 10.0 indicate moderate inflammation.^56^ In this study, CRP was collected from medical records.

SRDA was assessed using patient-reported outcome measures (PROMs) that are valid and reliable for the assessment of disease severity in IBD and widely used in IBD research.^57–59^ The PROMs show good-excellent accuracy, correctly classifying 80% to 90% of endoscopy-defined activity in UC,^57^ 79% to 91% of CD Activity Index (CDAI)-defined remission in CD.^58^ Displayed questions depended on disease subtype and presence of stoma. Questions pertained to abdominal pain severity over the last 7 days (answered by CD, UC, and those with a stoma), stool frequency over the last 7 days (answered by CD and UC), and rectal bleeding over the last 3 days (UC only). For descriptive statistics, the PROMs are reported separately by disease group. However, for inferential statistics, a z-score was calculated so that SRDA could be used as a single measure within the total sample. Here, Cronbach α=0.98 and 0.92-0.96 for CD and UC, respectively.

#### Secondary Outcomes

Secondary outcomes included self-reported disease information (eg, flare frequency and severity), quality of life, self-reported health care usage, and non-disease-specific biomarkers.

The non-disease-specific biomarkers of systemic inflammatory activity were those typically measured within routine phlebotomy in IBD. Specifically, these were counts of: neutrophils, lymphocytes, monocytes, and total white blood cells, as well as ferritin levels. These markers increase in response to inflammation and are higher when disease is active.^60–67^ In this study, these biomarkers were collected from medical records.

Secondary outcomes that were collected using self-reported questionnaires pertained to self-reported disease information, quality of life, and health care usage.

Self-reported disease information was captured using the following questions:

- Flare frequency in the last 3 months
- Severity of most recent flare (none, mild, moderate, or severe)
- The Patient Global Impression Scales of Severity (PGI-S) and the Patient Global Impression Scales of Improvement (PGI-I) assessed perceived illness symptomology,^68^ which ask about perceived physical symptom severity and improvement, respectively. The PGI-S is 1-item (range 0 to 3), collected at all 3 study timepoints. The PGI-I is 1-item (range 0 to 6). Higher scores indicate greater severity and greater deterioration, respectively. Both measures have demonstrated construct validity for incontinence, a common symptom in IBD.^68^ The PGI-I was planned to be collected at both 12 weeks and 6 months; however, due to a technical error it was not included in the 12-week questionnaire.

- The European Quality of Life Scale (EQ-5D-3L)^69^ measured health-related quality of life. It has 5 items on a 3-point scale assessing mobility, self-care, usual activities, pain/discomfort, and anxiety/depression. Item scores are inputted into an algorithm to give an index, which is used by the National Institute of Care and Excellence (NICE) to determine cost-effectiveness of interventions for approval in the NHS in the UK.^70^ Higher index scores indicate better quality of life. It is valid, reliable, and responsive to change in IBD populations.^71,72^ Here, Cronbach α=0.64 to 0.79.
- Questions adapted from the Client Service Receipt Inventory (CSRI),^73^ used in previous COMPASS evaluations,^42,46^ assessed health care use relating to GP visits, psychologist/therapists, emergency care, and secondary care. It has been shown to be valid and reliable (test-retest) in people living with long-term physical health conditions, such as IBD, demonstrating excellent (84.3%) agreement with hospital records.^74,75^ Questions asked frequency of use, and length of contact, allowing calculation of total minutes.

Psychological distress, measured with The Patient Health Questionnaire-Anxiety and Depression Scale (PHQ-ADS),^50^ was included as a mechanism variable. It is a combined score of the Patient Health Questionnaire (PHQ-9)^76^ for depression and the Generalised Anxiety Disorder Scale (GAD-7)^77^ for anxiety. It is a reliable and valid measure of distress in long-term physical health conditions, including IBD.^21,22^ Higher scores indicate greater distress (score ≥10 indicating clinically significant distress). In this study, Cronbach α=0.90 to 0.94.

### Statistical Analysis

A statistical analysis plan was published (https://osf.io/5yqm4/) on November 14, 2024.^78^ Data were analyzed with Stata Version 18. Descriptives were reported using (1) means and SDs for normal continuous data; (2) medians and IQRs or ranges for non-normal continuous data; (3) n and percentages for categorical data. Mixed-effect models were used to assess change in primary and secondary outcome variables from baseline to 12-week and 6-month follow-up (linear mixed-effects for continuous variables, proportional odds mixed-effects model for one ordinal variable), with a random intercept to account for repeated assessments within individuals. Assessment number (pre-intervention, 12 weeks, 6 months) was entered into models as a dummy-coded covariate, to estimate change from baseline, as well as adjustment for age and gender. Standardized mean differences (SMDs or Cohen *d*) and standardized response means (SRMs) quantified effect sizes for linear outcomes. Cutoffs for small, medium, and large effects were 0.2, 0.5, and 0.8, respectively.^79^ Odds ratios quantified effect size for ordinal outcomes. For the primary outcomes and psychological distress, change scores were calculated. For each timepoint, change scores in primary outcomes were correlated with changes in distress, before being inputted into multiple linear regression models, adjusting for age and gender.

The following sensitivity analyses were conducted:

1. Treatment effects for primary, secondary, and mechanism variables (aim 1) were conducted in the per-protocol sample. Adherence was defined as attending ≥3 therapist appointments and completing ≥5 online sessions.

In both the primary analyses (aim 1) and the analyses assessing relationships between changes in distress and IBD disease indicators (aim 2):

1. Participants who changed their medication throughout the trial were omitted to exclude those who may experience an improvement in inflammation and physical health due to medication changes.

1. For the biomarker data collected from medical records, a sensitivity analysis was run only including data collected within 1 month of a study timepoint. This differs from the study criteria, where data were eligible for inclusion if they were within 3 months of the study timepoint.

Mixed-effects and multiple linear regression models were assessed for violations of assumptions using Q-Q plots of residuals and scatterplots of fitted versus residual values. Where assumptions were violated, adjustments were made either by excluding extreme values/outliers or log-transforming variables, which is commonly done for analysis of biomarkers.^80^ Given the skew usually observed with CRP,^81^ it was log-transformed before analysis.

### Sample Size Calculation

The power calculations are detailed in the statistical analysis plan (https://osf.io/5yqm4/).^78^ In brief, a meta-analysis found that interventions for mood in IBD have effect sizes of *d*=0.19, *d*=0.29, and *d*=0.34 for fecal calprotectin, CRP, and general inflammation (including white blood cells and cytokines), respectively.^23^ Therefore, to achieve 80% power (α=0.05), the samples required for *n*=220 for FCP, *n*=96 for CRP, and *n*=70 for general inflammation.

## RESULTS

**FIGURE 1:**
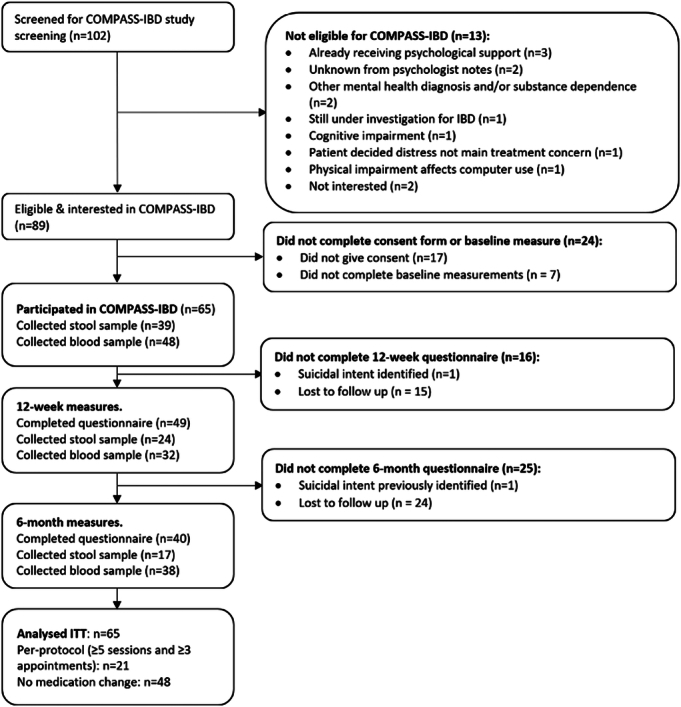
Flowchart of participants through the study.

Table 1 shows patient characteristics, including key demographics, medications, and disease activity. Other demographic, psychosocial, and engagement data are reported elsewhere.^49^ Follow-up rates were 75.4% at 12 weeks and 60.0% at 6 months (Figure 1). Group differences between participants where post-intervention questionnaire data were available versus unavailable are shown in Table S1, Supplemental Digital Content, http://links.lww.com/PSYMED/B153. Nonresponders had significantly higher distress (PHQ-ADS) and depression (PHQ-9) scores at baseline.

**TABLE 1:** Demographic and Clinical Characteristics of Participants in the COMPASS-IBD Study (*N*=65).

Demographic Characteristics	*N* (%)	M (SD)
Age (min= 18, max= 74)	—	36.49 (13.62)
Gender		
Male	26 (40)	—
Female	38 (58.5)	—
Other (trans female)	1 (1.5)	—
Ethnicity		
White	49 (75.4)	—
Mixed/multiple ethnic groups	3 (4.6)	—
Asian or Asian British	7 (10.8)	—
Black or Black British	4 (6.2)	—
Other ethnic groups	2 (3.1)	—
Index of multiple deprivation decile^a^ (min=1, max=10)	—	5.13 (2.38)
Employment status		
Employed	44 (67.6)	—
Retired		
Unemployed	4 (6.2)	—
Retired	4 (6.2)	—
Student	4 (6.2)	—
Long-term sick or disabled	5 (7.7)	—
Home-maker/carer	1 (1.5)	—
Not working and not on benefits	3 (4.6)	—

Clinical Characteristics	*N* (%)	M *(SD)*
IBD diagnosis		
Crohn disease	42 (64.4)	—
Ulcerative colitis	19 (29.2)	—
Indeterminate	3 (4.6)	—
Unsure	1 (1.5)	—
Years since IBD diagnosis (min= 0, max= 45)	—	9.67 (9.78)
Use of psychotropic medication		
Prescribed and taking	18 (27.7)	—
Prescribed and not taking	6 (9.2)	—
Not prescribed	40 (61.5)	—
Unsure	1 (1.5)	—
Medications		
Aminosalicylates/5ASA	20 (30.8)	
Thiopurines	23 (35.4)	
Methotrexate	3 (4.6)	
Biologics	32 (49.2)	
Steroids	10 (15.4)	
No IBD medication	14 (21.5)	
Stoma		
Yes	7 (10.8)	
No	58 (89.2)	
Fistula		
Yes	6 (9.2)	
No	49 (75.4)	
Unsure	10 (15.4)	
Flares in last 2 y		
0	7 (10.8)	
1-2	11 (16.9)	
3-4	12 (18.5)	
≥5	23 (35.4)	
Unsure	12 (18.5)	
Smoking status		
Never smoked	41 (63.1)	
Former Smoker	17 (26.2)	
Current smoker	7 (10.8)	
History of IBD operations		
Yes	24 (36.9)	
No	41 (63.1)	
Anxiety (GAD-7)		11.60 (5.07)
Depression (PHQ-9)		11.95 (6.23)
Distress (PHQ-ADS)		23.55 (10.23)
Quality of life (EQ5D)		0.70 (0.19)
SRDA (CD) (*n*=36, min=0, max=10.6)		3.86 (3.15)
SRDA (UC) (*n*=19, min=1, max=8.3)		3.56 (2.19)
SRDA (stoma) (*n*=5, min=0.4, max=3)		1.43 (1.28)

IBD=inflammatory bowel disease; GAD-7=Generalised Anxiety Disorder Scale; PHQ-9=Patient Health Questionnaire-9 Scale; PHQ-ADS=Patient Health Questionnaire Anxiety and Depression Scale; EQ5D=European Quality of Life Scale; CD=Crohn disease; UC=ulcerative colitis; GP=general practitioner; PGIS=Patient Global Impression Scales of Severity; SRDA=self-reported disease activity.

^a^ *n*=60 as 5 participants’ postcodes had no SES data available.

### Primary and Secondary Effectiveness Outcomes

The adjusted treatment effects of COMPASS-IBD for the primary outcomes (disease indicators) and secondary outcomes (additional inflammatory biomarkers, self-reported disease information, and self-reported health care usage) are shown in Table 2.

**TABLE 2:** Treatment Effect Estimates and Standardized Mean Difference for Effectiveness Adjusted for Age and Gender

	Time	*n*	Mean (SD)	*B*	SE	95% CI	*P*	Cohen *d*	SRM
Fecal calprotectin (μg/g)	1	39	491.10 (1010.73)						
	2	24	187.17 (376.52)	−316.61	184.53	−678.28, 45.06	0.086	−0.378	−0.269
	3	17	115.53 (161.66)	−383.07	205.22	−787.41, 21.77	0.064	−0.444	−0.420
CRP (mg/L)^a^	1	48	7.21 (25.83)						
	2	32	4.25 (9.11)	−0.753	0.265	−1.27, 0.23	0.005	−0.528	−0.498
	3	38	2.16 (3.29)	−0.674	0.251	−1.17, −0.18	0.007	−0.466	−0.393
SRDA	1	60	0.037 (1.069)						
	2	34	0.113 (0.909)	−0.004	0.152	−0.30, 0.29	0.977	−0.004	−0.004
	3	29	−0.208 (0.917)	−0.291	0.163	−0.61, 0.03	0.075	−0.284	−0.361
WBC (cells/μL)	1	50	7.49 (2.43)						
	2	35	6.59 (2.04)	−0.823	0.33	−1.56, −0.12	0.012	−0.356	−0.421
	3	40	6.92 (2.47)	−0.744	0.32	−1.23, −0.16	0.020	−0.319	−0.339
Ferritin (µg/L)	1	27	89.96 (256.22)						
	2	11	123.82 (128.34)	21.34	70.98	−119.25, 161.93	0.766	0.095	0.041
	3	10	82.8 (109.14)	7.15	74.17	−138.21, 152.52	0.923	0.032	0.315
Lymphocytes (cells/nL)	1	50	1.91 (0.83)						
	2	35	1.93 (1.06)	0.087	0.10	−0.11, 0.29	0.392	0.094	0.129
	3	41	2.1 (1.13)	0.176	0.10	−0.01, 0.37	0.070	0.180	0.329
Monocytes (cells/nL)	1	50	0.521 (0.162)						
	2	35	0.541 (0.19)	0.025	0.024	−0.02, 0.07	0.300	0.146	0.167
	3	41	0.562 (0.179)	0.045	0.023	0.00, 0.09	0.053	0.265	0.281
Neutrophils (cells/nL)	1	50	4.73 (2.27)						
	2	35	3.92 (1.34)	−0.794	0.34	−1.45, −0.13	0.018	−0.401	−0.387
	3	41	4.14 (1.85)	−0.759	0.32	−1.39, −0.13	0.019	−0.384	−0.394
Flare frequency	1	65	3.32 (1.67)						
	2	44	2.36 (1.94)	−0.95	0.30	−1.54, −0.35	0.002	−0.515	−0.382
	3	34	1.81 (1.78)	−1.49	0.33	−2.14, −0.83	<0.001	−0.804	−0.722
Flare severity^b^	1	64	None: 7 (10.94%) Mild: 8 (12.50%) Moderate: 27 (42.19%) Severe: 22 (34.38%)						
	2	40	None: 9 (22.50%) Mild: 6 (15.00%) Moderate: 16 (40.00%) Severe: 9 (22.50%)	OR=0.42	0.17	0.19, 0.95	0.036		
	3	32	None: 9 (28.12%) Mild: 7 (21.88%) Moderate: 8 (25.00%) Severe: 8 (25.00%)	OR=0.34	0.16	0.14, 0.84	0.019		
GP usage (min)	1	65	36.9 (43.41)						
	2	41	8.34 (9.32)	−30.31	9.13	−48.21, −12.41	0.001	−0.817	−0.650
	3	34	53.19 (84.95)	14.82	9.76	−4.31, 33.96	0.129	0.243	0.174
Psychological support (min)	1	65	53.3 (113.88)						
	2	41	17.56 (44.99)	−37.41	15.5	−67.86, −6.95	0.016	−0.395	−0.309
	3	34	7.94 (26.69)	−46.50	16.6	−78.94, −13.99	0.005	−0.485	−1.590
A&E usage (min)	1	65	873.38 (3162)						
	2	41	22.14 (75.08)	−841.67	424.12	−1672.9, −10.4	0.047	−0.336	−0.254
	3	34	3.33 (9.6)	−900.95	450.64	−1784.2, −17.7	0.046	−0.348	−15.970
Secondary care usage (min)	1	65	229.34 (1262.52)						
	2	41	18.05 (44.34)	−210.70	169.24	−542.41, 121.02	0.213	−0.213	−0.814
	3	34	55.7 (105.11)	−187.36	179.83	−539.82, 165.09	0.297	−0.183	−1.759
Health-related quality of life (EQ5D)	1	65	0.697 (0.194)						
	2	41	0.743 (0.171)	0.032	0.018	−0.00, 0.07	0.079	0.172	0.273
	3	34	0.728 (0.172)	0.027	0.020	−0.01, 0.07	0.167	0.145	0.248
Global Symptom Impression (PGIS)	1	65	2.25 (0.97)						
	2	41	2.1 (1.02)	−0.13	0.14	−0.39, 0.14	0.350	−0.129	−0.127
	3	33	2.15 (0.83)	−0.09	0.15	−0.38, 0.20	0.559	−0.094	−0.122
Distress (PHQ-ADS)	1	65	23.55 (10.24)						
	2	49	16.19 (11.26)	−6.25	1.12	−8.45, −4.06	<0.001	−0.556	−0.657
	3	40	17.03 (10.85)	−6.70	1.21	−9.07, −4.33	<0.001	−0.615	−0.823

SRM=standardized response mean; CRP=C-Reactive Protein; SRDA=self-reported disease activity; WBC=white blood cell count; GP=general practitioner; A&E=accident and emergency; EQ5D=European Quality of Life Scale; PGIS=Patient Global Impression Scales of Severity; PHQ-ADS=Patient Health Questionnaire Anxiety and Depression Scale.

The PGII was listed as an effectiveness outcome; however, due to technical issues with the survey software, it was not collected. Twelve-week data presented only for those who responded (missing completely at random, MCAR, assumption), even though the mixed model is based on a missing at random, MAR, assumption. Models used maximum likelihood estimation for missing data.

^a^ Mean and SD column show raw values, although due to issues with skew, the log-transform was used in the mixed-effects models, in line with the statistical analysis plan. Unstandardized estimates, standardised error, and 95% CIs relate to the log-transform value.

^b^ Ordinal variable analyzed with proportional odds mixed-effects model that estimated odds ratios instead of unstandardised beta.

Assumptions were violated for the following variables: FCP, WBC, ferritin, lymphocytes, monocytes, neutrophils, GP usage, psychological support, A&E, secondary care usage, and EQ-5D. After adjustments, the models abided by the assumptions without substantive changes to SMD or *p*-values; therefore, the statistics from the original models are reported.

For the primary outcomes, results demonstrated small-moderate, albeit nonsignificant, effect sizes for FCP at both follow-up timepoints (12 wk: *d*=−0.378, *P*=0.086; 6 mo *d*=−0.444, *P*=.064), with estimated reductions of 316.61 μg/g (95% CI: −678.3, 45.1) and 383.07 μg/g (95% CI: −785.3, 19.2), respectively. CRP was significantly reduced at both time points, yielding small-to-moderate effect sizes (12 wk: *d*=−0.528, *P*=.005; 6 mo: *d*=−0.466, *P*=.007). SRDA had a nonsignificant negligible effect at 12 weeks (*d*=−0.004, *P*=.977), but showed a small nonsignificant effect at 6 months (*d*=−0.284, *P*=.075).

Secondary outcomes included additional inflammatory markers, self-reported disease information (eg, flare frequency and severity), and self-reported health care usage (Table 2). Regarding inflammatory markers, significant reductions were seen at both timepoints for white blood cell (12 wk: *B*=−0.823, 95% CI: −1.47, −0.18, *P*=.012, *d*=−0.356; 6 mo: *B*=−0.744, 95% CI: −1.37, −0.12, *P*=.020, *d*=−0.319) and neutrophil concentrations (12 wk: *B*=−0.794, 95% CI: −1.45, −0.13, *P*=.018, *d*=−0.401; 6 mo: *B*=−0.759, 95% CI: −1.45, −0.13, *P*=.018, *d*=−0.384). Monocytes showed a significant increase at 6 months (*B*=0.045, 95% CI: 0.00 – 0.09, *d*=0.265, *P*=.053), but not at 12 weeks (*B*=0.025, 95% CI: −0.02 – 0.07, *d*=0.300, *P*=.146). There were no significant changes in lymphocytes or ferritin levels.

For self-reported health outcomes, participants reported significant moderate-large reductions in flare frequency, with participants experiencing 0.95 fewer flares (95% CI: −1.4, −0.4, *d*=−0.515, *P*=.002) in the last 3 months at 12 weeks, and 1.49 fewer flares (95% CI: −2.1, −0.8, *d*=−0.804, *P*<.001). Moreover, participants reported 58% lower odds of experiencing a higher flare severity score at 12 weeks (OR=0.42, *P*=.036, 95% CIs: 0.19, 0.95), and 66% lower odds at 6-month follow-up (OR=0.34, *P*=.019, 95% CIs: 0.14, 0.84). However, there were no significant reductions in health-related quality of life or perceived illness symptomology.

Participants demonstrated significantly reduced health care usage, with small, significant effects on psychological health care professional usage (12 wk: *B*=−37, 95%CI: −68, −7, *d*=−0.395, *P*=.016; 6 mo: *B*=−47, 95% CI: −79, −14, *d*=−0.495, *P*=.005) and A&E usage (12 wk: *B*=−842, 95% CI: −1673, −10, *d*=−0.336, *P*=.047; 6 mo: *B*=−901, 95% CI: −1784, −18, *d*=−0.348, *P*=.046). There was a large effect for GP usage at 12 weeks (*B*=−30, 95% CI: −48, −12, *d*=−0.817, *P*=.001), although this was not sustained at 6 months. There was no reduction in secondary care usage.

### Relationships Between Changes in Distress and IBD Disease Indicators

To address the second study aim, the relationships between changes in primary outcomes and changes in distress were assessed. Psychological distress demonstrated significant reductions post-intervention (*B*=−6.25, 95% CI: −8.4, −4.1, *d*=−0.556, *P*<.001) and at 6-months follow-up (*B*=−6.70, 95% CI: −9.1, −4.3, *d*=−0.615, *P*<.001) (Table S2, Supplemental Digital Content, http://links.lww.com/PSYMED/B153). Subsequently, correlations and multiple linear regressions adjusted for age and gender were conducted. Correlation coefficients are shown in Table S2, Supplemental Digital Content, http://links.lww.com/PSYMED/B153. Changes in FCP and CRP were not significantly associated with changes in distress. Changes in SRDA were associated with changes in distress at 12 weeks (*r*=0.399, *P*=.05), but there was no significant association at 6 months.

The regression models at both timepoints for FCP were violated. After the models were adjusted and assumptions were met, the size and direction of B values changed substantially, so statistics from these models are reported in Table S2, Supplemental Digital Content, http://links.lww.com/PSYMED/B153. In the regression models, change in distress did not significantly predict change in any of the primary outcomes, at either timepoint.

### Sensitivity Analyses

The first sensitivity analysis was a per-protocol analysis, conducted for primary and secondary outcomes. A total of 43 participants (66.2%) were adherent to the intervention (≥3 therapist appointments, ≥5 online sessions). The effects were generally consistent with the intention-to-treat (ITT) analysis, and were occasionally slightly larger (Table S3, Supplemental Digital Content, http://links.lww.com/PSYMED/B153), for instance there was a moderate 6-month effect for CRP in the per-protocol sample (*d*=−0.514, *P*=.03) compared with a small effect in the ITT sample (*d*=−0.276, *P*=.05).

The second sensitivity analysis re-ran mixed models, omitting participants who self-reported changes to their medication throughout the trial. Effect sizes were largely consistent with the ITT sample (Table S4, Supplemental Digital Content, http://links.lww.com/PSYMED/B153). Likewise, there were marginal differences in correlation and regression coefficients in the analysis investigating relationship with changes in psychological distress (Table S5, Supplemental Digital Content, http://links.lww.com/PSYMED/B153).

The third sensitivity analysis analyzed biomarkers, but imposed a stricter time limit between sample and study timepoint (1 mo instead of 3 mo). This greatly reduced the number of observations, which prohibited analysis of FCP and ferritin. Table S6, Supplemental Digital Content, http://links.lww.com/PSYMED/B153, shows that effect sizes were generally smaller, but significant reductions were still observed for white blood cells (*d*=−0.275, *P*=.05) and neutrophils (*d*=−0.313, *P*=.05). The relationship analysis could only be conducted for CRP. With the stricter limit imposed, changes in CRP at 6 months showed a moderate positive correlation with changes in distress (*r*=0.596, *P*=.05). Change in distress significantly positively predicted change in CRP at 6 months (*B*=5.284, SE=2.18, 95% CIs: 0.1, 10.4, *P*=.046, see Table S7, Supplemental Digital Content, http://links.lww.com/PSYMED/B153).

## DISCUSSION

This exploratory pilot study evaluated whether COMPASS-IBD, a digital CBT intervention tailored to IBD, affected disease activity and broader health outcomes. The results suggest potentially meaningful reductions in systemic inflammation, flare frequency and severity, and health care use. However, the nonrandomised design means results must be interpreted cautiously.

Results were mixed for the 3 primary outcomes. For CRP, there were significant small-moderate reductions at both timepoints (*d* ranged from −0.47 to −0.53, *P*<.01), whereas FCP showed small nonsignificant effect sizes at both timepoints (*d* ranged from −0.38 to −0.44, *P*<.10). SRDA demonstrated no change at 12 weeks, however, there was a trend for improved SRDA at 6 months (*d*=−0.28, *P*=.08). Analysis of secondary outcomes revealed significant small-moderate reductions in white blood cells and neutrophils, alongside reduced flare frequency and severity. Participants reported reduced health care usage after COMPASS-IBD relating to GPs, psychologists and A&E, but not secondary care.

This study extends the implementation paper,^49^ demonstrating that reductions in psychological distress observed at 12 weeks were maintained at 6-month follow-up. The covariation of distress and primary outcomes (FCP, CRP, and SRDA) was largely nonsignificant. This may be explained by disease activity improving independent of the intervention, due to changes to medications and/or treatments. However, a sensitivity analysis was run to assess this risk, demonstrating no discernible differences in effect size or direction. Therefore, the null findings may have been due to methodological aspects of the study, such as low power and asynchronous biomarker and questionnaire data collection. Although the utilization of routinely collected biological data in this study has high translational relevance, analyses may have been compromised. Indeed, when a stricter time limit was imposed, improvements in distress were positively moderately correlated with CRP, suggesting that future trials should align physiological and self-report measures.

The size of the CRP reduction (from 7.21 to 2.16 mg/L) is clinically notable. Concentrations exceeding ≥3 mg/L confer increased risk for cardiovascular and metabolic conditions,^82,83^ and individuals with IBD already face increased risk of developing these conditions.^84–86^ Metabolic complications can worsen disease course,^87^ potentially due to shared inflammatory pathways.^84,88,89^ The reduction in CRP is also compelling, as elevated CRP has been associated with greater antidepressant resistance.^90^ Confirming this dual benefit in a RCT would strengthen the clinical rationale for COMPASS-IBD.

Although FCP levels were not significantly reduced, this may reflect methodological limitations rather than a null effect. First, effect sizes were consistent with a meta-analysis of interventions for distress on IBD activity.^23^ Second, the sample was underpowered to detect small-moderate effects, as specified in the statistical analysis plan^78^ (link: https://osf.io/5yqm4/). Third, sensitivity analyses that excluded participants who changed their medication showed no substantive changes compared with the complete sample. Given the promising pilot data, a fully powered RCT should confirm whether COMPASS-IBD can reliably influence inflammatory disease activity.

Despite mixed findings for the SRDA measure, the frequency and severity of self-reported flares were significantly reduced at both timepoints. Indeed, improvements in SRDA correlated with changes in psychological distress. These findings contradict a previous meta-analysis indicating no effect of psychological interventions on IBD disease activity indices.^38^ This discrepancy may reflect distinctive features of COMPASS-IBD, built on the TMA-LTC, including modules that target symptom perception and self-management.^41,42^ Another reason for disagreements in the literature could be individual differences in gut-brain mechanisms. For instance, in UC, microbial and metabolic profiles have been linked to stress reactivity and subsequent clinical flare risk;^91^ therefore, psychological interventions may only be effective at reducing inflammation and risk of flares in certain sub-groups of patients.

Although the intervention showed tentative signals of reduced inflammation, the decline in health care utilization was more notable. Interestingly, the main analysis of this trial showed that participants had improved self-efficacy after the intervention.^49^ Reduced service use may be driven by changes in help-seeking behavior, rather than by direct improvements in symptoms or inflammation. The health care outcomes observed here corroborate an RCT of the transdiagnostic COMPASS program,^46^ showing that the IBD-tailored version can reduce service use when delivered in a real-world NHS setting. Past research demonstrates that psychological care in IBD services can reduce health care costs.^33,34^ Digital therapy requires less clinician time and offers greater flexibility^37,92^ so programs like COMPASS-IBD may be more scalable and acceptable within IBD care.^36^

### Limitations and Future Directions

Several limitations must be acknowledged. The small, nonrandomised sample limited statistical power for biomarker analyses. Adherence to COMPASS-IBD was suboptimal, which may have diluted effects. Opportunistic extraction of biomarker data may have introduced variability in sampling intervals. Although sensitivity analyses attempted to mitigate this, causal inference remains restricted. This approach took advantage of routine clinical monitoring, enabling multiple biomarkers to be assessed in a cost-effective way while maintaining translational relevance. Future research should systematize biomarker data collection over longer timeframes to understand how stable these are without intervention, and explore the potential mediating gut-brain axis mechanisms which may underlie improvements in disease indicators. These may include behavioral changes (eg, improved sleep or medication adherence), altered autonomic reactivity, and microbial changes. Questionnaire completion was 84.6% at 12 weeks and 60.0% at 6 months, with lower response among participants reporting greater baseline depression. This may falsely inflate intervention effects; however, the mixed-effects models handle missing data with a minimally restrictive assumption (Missing At Random, MAR). The use of objective measures of inflammation alongside self-report aimed to give a holistic picture of IBD disease activity. This was deemed necessary given the well-reported differences in inflammation and self-reported symptoms, and the psychosocial predictors of health care usage.^20,24–27,30,93,94^

## CONCLUSIONS

This pilot study provides preliminary evidence that COMPASS-IBD, a digital CBT intervention for IBD patients, has the potential to reduce disease indicators, inflammatory biomarkers, and health care usage, alongside psychological distress. A larger sample utilizing a randomized design is needed to confirm these findings. Moreover, mediation analyses would help to elucidate gut-brain mechanisms underlying the promising effects and help to enhance or personalize interventions to boost treatment effects. The study underscores the value of including scalable and effective psychological treatments, as part of integrated care in IBD management.

## Supplementary Material
