# Supplementary material for: A Pilot Study of the Real-World Impact of Digital Cognitive Behavioral Therapy for Inflammatory Bowel Disease (COMPASS-IBD) on Inflammation, Disease Activity, and Healthcare Use
Source: Biopsychosoc Sci Med. 2025 Nov 21;88(3):274–86. doi: 10.1097/PSY.0000000000001457 (PMC13043230; doi:10.1097/PSY.0000000000001457)
Supplement: Supplementary file 1 [file psy-88-274-s001.docx]

Supplementary Material

**Table S1**. Baseline characteristics between those available in ITT analysis and those with no post-intervention data

| **Demographic characteristics** | | **Available for analysis** | **No post-intervention data** | **p value** |
| --- | --- | --- | --- | --- |
|  | | **N=55** | **N=10** |  |
|  | |  |  |  |
| **Age (M, SD)** | | 35.78 (13.60) | 40.40 (13.80) | 0.328 |
| **Gender (n, %)** | |  |  |  |
|  | % Female | 34 (61.8%) | 5 (50.0%) | 0.483 |
| **Ethnicity (n, %)** | |  |  |  |
|  | % Non-minority | 40 (72.7%) | 9 (90.0%) | 0.243 |
| **Index of Multiple Deprivation Decile^$^** | | 5.17 (2.40) | 4.89 (2.37) | 0.741 |
| **Employment status (n, %)** | |  |  | 0.538 |
|  | % Employed | 42 (76.4%) | 6 (60.0%) |  |
|  | % Unemployed | 9 (16.4%) | 3 (30%) |  |
|  | % Retired | 4 (7.3%) | 1 (10%) |  |
| **Education (n, %)** | |  |  |  |
|  | % ≥Degree level | 41 (74.6%) | 6 (60.0%) | 0.344 |
| **IBD diagnosis (n, %)** | |  |  |  |
|  | % Crohn’s Disease | 35 (63.6%) | 7 (70%) | 0.150 |
| **Years since IBD diagnosis (M, SD)** | | 9.33 (8.80) | 11.50 (14.48) | 0.524 |
| **Stoma (n, %)** | |  |  | 0.932 |
|  | % Yes | 6 (10.9%) | 1 (10.0%) |  |
| **Fistula (n, %)** | |  |  | 0.897 |
|  | % Yes | 42 (76.4%) | 7 (70%) |  |
|  | % No | 5 (9.1%) | 1 (10.0%) |  |
|  | % Unsure | 8 (14.6%) | 2 (20.0%) |  |
| **Flares in last 2 years (M, SD)** | | 3.20 (1.89) | 4.00 (1.60) | 0.266 |
| **Smoking status** | |  |  |  |
|  | % Never smoked | 37 (67.3%) | 4 (40.0%) | 0.077 |
| **IBD operations** | |  |  |  |
|  | % Yes | 21 (38.2%) | 3 (30.0%) | 0.622 |
| **Distress** (PHQ-ADS) | | 22.42 (9.82) | 29.80 (10.73) | 0.035 |
| **Anxiety** (GAD-7) | | 11.31 (5.01) | 13.20 (5.37) | 0.281 |
| **Depression** (PHQ-9) | | 11.11 (5.99) | 16.60 (5.89) | 0.009 |
| **Quality of life** (EQ-5D) | | 0.71 (0.18) | 0.62 (0.24) | 0.197 |
| **SRDA** (Z score) | | 0.01 (1.07) | 0.21 (1.12) | 0.592 |

^$^ participants available n=51, participants not available n=9, (total n=60) as 5 participants’ postcodes had no SES data available. EQ5D= European Quality of Life Scale, GAD-7=Generalised Anxiety Disorder Scale, IBD=inflammatory bowel disease, PGIS= Patient Global Impression Scales of Severity, PHQ-ADS=Patient Health Questionnaire Anxiety and Depression Scale, PHQ-9=Patient Health Questionnaire-9 Scale, SRDA=self-reported disease activity

**Table S2.** Correlations and regressions examining the relationship between changes in primary outcomes and changes in psychological distress

|  |  | *n* | Correlation |  | Regression |  |  |  |
| --- | --- | --- | --- | --- | --- | --- | --- | --- |
|  |  |  | *r* |  | *B* | SE | 95%CI | *p* value |
| Faecal Calprotectin | 1 | 17 | -0.041 |  | 4.361 | 3.9 | -4.3, 13 | .30 |
|  | 2 | 10 | 0.091 |  | 1.627 | 4.2 | -7.9, 11.1 | .71 |
| CRP | 1 | 25 | -0.377 |  | -0.091 | 0.09 | -0.3, 0.1 | .30 |
|  | 2 | 22 | 0.116 |  | -0.001 | 0.04 | -0.09, 0.1 | .99 |
| SRDA | 1 | 33 | -0.013 |  | 0.014 | 0.01 | -0.02, 0.04 | .34 |
|  | 2 | 27 | 0.392* |  | -0.032 | -0.02 | -0.08, 0.01 | .13 |

CRP=C-Reactive Protein, SRDA=self-reported disease activity

**Table S3**. Treatment effect estimates and standardised mean difference for effectiveness adjusted for age and gender in the per-protocol sample

|  | Time | *n* | Mean (SD) | *B* | SE | 95% CI | *p* | Cohen’s *d* |
| --- | --- | --- | --- | --- | --- | --- | --- | --- |
| Faecal calprotectin | 1 | 18 | 243.33 (743.94) |  |  |  |  |  |
|  | 2 | 8 | 41.5 (74.45) | -0.775 | 0.48 | -1.72, 0.17 | .108 | -0.479 |
|  | 3 | 6 | 36.83 (52.07) | -0.756 | 0.48 | -1.7, 0.19 | .117 | -0.455 |
| CRP | 1 | 15 | 2 (1.51) |  |  |  |  |  |
|  | 2 | 12 | 1.33 (1.15) | -0.407 | 0.33 | -1.1, 0.2 | .522 | -0.129 |
|  | 3 | 12 | 2.5 (5) | 0.728 | 0.34 | -1.4, -0.1 | .299 | -0.225 |
| SRDA | 1 | 21 | -0.15 (0.96) |  |  |  |  |  |
|  | 2 | 16 | 0.03 (0.73) | 0.194 | 0.18 | -0.2, 0.6 | .287 | 0.225 |
|  | 3 | 12 | -0.43 (0.69) | -0.183 | 0.2 | -0.6, 0.2 | .370 | -0.209 |
| WBC | 1 | 16 | 7.23 (2.29) |  |  |  |  |  |
|  | 2 | 12 | 5.84 (2.17) | -0.89 | 0.48 | -1.8, 0.1 | .065 | -0.385 |
|  | 3 | 12 | 6.13 (1.97) | -0.72 | 0.48 | -1.7, 0.2 | .135 | -0.328 |
| Ferritin* | 1 | 6 | 15 (8.51) |  |  |  |  |  |
|  | 2 | 3 | 88.33 (131.37) |  |  |  |  |  |
|  | 3 | 2 | 57 (70.71) |  |  |  |  |  |
| Lymphocytes | 1 | 16 | 2.09 (0.92) |  |  |  |  |  |
|  | 2 | 12 | 1.79 (0.96) | -0.056 | 0.13 | -0.3, 0.2 | .676 | -0.060 |
|  | 3 | 13 | 2.06 (1.18) | 0 | 0.13 | -0.3, 0.3 | .999 | -0.000 |
| Monocytes | 1 | 16 | 0.56 (0.15) |  |  |  |  |  |
|  | 2 | 12 | 0.46 (0.12) | -0.045 | 0.03 | -0.1, 0.0 | .120 | -0.302 |
|  | 3 | 13 | 0.54 (0.23) | -0.007 | 0.03 | -0.1, 0.0 | .798 | -0.037 |
| Neutrophils^£^ | 1 | 16 | 4.13 (2.11) |  |  |  |  |  |
|  | 2 | 12 | 3.39 (1.41) | -0.178 | 0.10 | -0.4, 0.0 | .083 | -0.443 |
|  | 3 | 13 | 3.86 (1.6) | -0.136 | 0.10 | -0.3, 0.1 | .185 | -0.339 |
| Flare frequency | 1 | 21 |  |  |  |  |  |  |
|  | 2 | 19 |  |  |  |  | .184 | -0.392 |
|  | 3 | 13 |  |  |  |  | **.011** | -0.861 |
| Flare severity^$^ | 1 | 21 | None: 2 (9.52%)  Mild: 3 (14.29%)  Moderate: 9 (42.86%)  Severe: 7 (33.33%) |  |  |  |  |  |
|  | 2 | 18 | None: 5 (27.78%)  Mild: 4 (22.22%)  Moderate: 6 (33.33%)  Severe: 3 (16.67%) | OR=0.23 | 0.16 | 0.1, 0.9 | **.032** |  |
|  | 3 | 12 | None: 4 (33.33%)  Mild: 2 (16.67%)  Moderate: 2 (16.67%)  Severe: 4 (33.33%) | OR=0.41 | 20.33 | 0.1, 1.9 | .261 |  |
| GP usage (minutes) ^£^ | 1 | 21 | 46.78 (55.01) |  |  |  |  |  |
|  | 2 | 19 | 6.73 (7.75) | -40.893 | 9.91 | -60.3, -21.5 | **<.001** | -0.917 |
|  | 3 | 13 | 19.97 (37.19) | -26.314 | 11.3 | -48.5, -4.2 | **.020** | -0.525 |
| Psychological support (minutes) ^£^ | 1 | 21 | 79.15 (148.55) |  |  |  |  |  |
|  | 2 | 19 | 11.05 (28.65) | -69.036 | 29.08 | -126, -12 | **.015** | -0.608 |
|  | 3 | 13 | 0 (0) | -81.157 | 32.61 | -145.1, -17.2 | **.016** | -0.665 |
| A&E usage (minutes) | 1 | 21 | 29.52 (122.25) |  |  |  |  |  |
|  | 2 | 19 | 24.42 (74.98) | -4.333 | 26.99 | -57.2, 48.6 | .872 | -0.043 |
|  | 3 | 13 | 2.05 (7.4) | -30.491 | 30.26 | -89.8, 28.8 | .314 | -0.317 |
| Secondary care usage (minutes) ^£^ | 1 | 21 | 124.23 (364.64) |  |  |  |  |  |
|  | 2 | 19 | 27.37 (62.17) | -98.279 | 62.92 | -221.6, 25.1 | .118 | -0.365 |
|  | 3 | 13 | 44.33 (102.63) | -84.24 | 71.68 | -224.7, 56.3 | .240 | -0.287 |
| Health related quality of life (EQ5D) | 1 | 21 | 0.76 (0.12) |  |  |  |  |  |
|  | 2 | 19 | 0.81 (0.11) | 0.043 | 0.02 | 0.0, 0.1 | .070 | 0.359 |
|  | 3 | 13 | 0.75 (0.16) | -0.002 | 0.02 | -0.1, 0.1 | .954 | -0.011 |
| Global Symptom Impression (PGIS) | 1 | 21 | 2.1 (0.89) |  |  |  |  |  |
|  | 2 | 19 | 1.89 (0.88) | -0.182 | 0.17 | -0.5, 0.2 | .303 | -0.207 |
|  | 3 | 13 | 1.85 (0.69) | -0.139 | 0.2 | -0.5, 0.3 | .491 | -0.171 |
| Distress (PHQ-ADS) | 1 | 21 | 23.38 (9.86) |  |  |  |  |  |
|  | 2 | 20 | 15 (11.23) | -8.499 | 1.93 | -12.3, -4.7 | <.001 | -0.755 |
|  | 3 | 13 | 16.15 (8.88) | -8.788 | 2.26 | -13.2, -4.3 | <.001 | -0.877 |

*Note*. ^£^ The mixed effects model assumptions were violated, however, once adjusted, there were minimal changes in B values, and effect sizes, so results from original models are presented.

^$^ ordinal variable analysed with proportional odds mixed-effects model that estimated odds ratios instead of unstandardised beta.

A&E=accident and emergency, CRP= C-Reactive Protein, GP = general practitioner, PGIS= Patient Global Impression Scales of Severity, PHQ-ADS=Patient Health Questionnaire Anxiety and Depression Scale, SRDA=self-reported disease activity, SRM=standardised response mean, WBC=white blood cell count.

**Table S4.** Treatment effect estimates and standardised mean difference for effectiveness adjusted for age and gender in a sensitivity analysis excluding participants with recent changes to medication

|  | Time | *n* | Mean (SD) | *B* | SE | 95% CI | *p* | Cohen’s *d* |
| --- | --- | --- | --- | --- | --- | --- | --- | --- |
| Faecal calprotectin^£^ | 1 | 29 | 578.07 (1114.2) |  |  |  |  |  |
|  | 2 | 17 | 250.76 (434.34) | -361.592 | 243.5 | -838.9, 115.8 | .138 | -0.389 |
|  | 3 | 12 | 142.75 (184.03) | -420.251 | 273.4 | -956.2, 115.7 | .124 | -0.438 |
| CRP | 1 | 35 | 8.97 (30.16) |  |  |  |  |  |
|  | 2 | 20 | 6.05 (11.2) | -0.123 | 0.20 | -0.5, 0.26 | .532 | -0.112 |
|  | 3 | 25 | 12.2 (46.78) | -0.227 | 0.19 | -0.6, 0.2 | .240 | -0.216 |
| SRDA | 1 | 44 | 0.11 (1.09) |  |  |  |  |  |
|  | 2 | 25 | 0.05 (0.94) | -0.066 | 0.18 | -0.4, 0.3 | .724 | -0.064 |
|  | 3 | 16 | -0.08 (0.94) | -0.261 | 0.22 | -0.7, 0.2 | .246 | -0.250 |
| WBC | 1 | 35 | 7.48 (2.6) |  |  |  |  |  |
|  | 2 | 22 | 6.33 (1.76) | -1.024 | 0.43 | -1.8, -0.2 | **.017** | -0.433 |
|  | 3 | 27 | 7 (2.65) | -0.761 | 0.41 | -1.6, 0.0 | .063 | -0.310 |
| Ferritin* | 1 | 22 | 95.14 (283.22) |  |  |  |  |  |
|  | 2 | 10 | 130.8 (133.06) |  |  |  |  |  |
|  | 3 | 7 | 64.14 (109.24) |  |  |  |  |  |
| Lymphocytes | 1 | 35 | 1.86 (0.87) |  |  |  |  |  |
|  | 2 | 22 | 1.7 (0.9) | 0.005 | 0.11 | -0.2, 0.2 | .965 | 0.006 |
|  | 3 | 28 | 2.08 (1.17) | 0.214 | 0.1 | 0, 0.4 | **.042** | 0.211 |
| Monocytes | 1 | 35 | 0.53 (0.18) |  |  |  |  |  |
|  | 2 | 22 | 0.55 (0.2) | 0.027 | 0.03 | 0, 0.1 | .415 | 0.148 |
|  | 3 | 28 | 0.58 (0.19) | 0.047 | 0.03 | 0, 0.1 | .128 | 0.260 |
| Neutrophils^£^ | 1 | 35 | 4.75 (2.44) |  |  |  |  |  |
|  | 2 | 22 | 3.9 (1.19) | -0.893 | 0.5 | -1.9, 0.1 | .079 | -0.430 |
|  | 3 | 28 | 4.32 (2.06) | -0.424 | 0.47 | -1.3, 0.5) | .366 | -0.187 |
| Flare frequency | 1 | 48 | 3.35 (1.6) |  |  |  |  |  |
|  | 2 | 31 | 2.63 (2.04) | -0.706 | 0.32 | -1.3, -0.1 | **.031** | -0.390 |
|  | 3 | 20 | 2.16 (1.74) | -1.284 | 0.38 | -2.0, -0.5 | **.001** | -0.746 |
| Flare severity^$^ | 1 | 48 | None: 4 (8.33%)  Mild: 5 (10.42%)  Moderate: 21 (43.75%)  Severe: 18 (37.50%) |  |  |  |  |  |
|  | 2 | 29 | None: 6 (20.69%)  Mild: 2 (6.90%)  Moderate: 15 (51.72%)  Severe: 6 (20.69%) | OR=0.325 | 0.17 | 0.1, 0.9 | **.036** |  |
|  | 3 | 19 | None: 3 (15.79%)  Mild: 5 (26.32%)  Moderate: 6 (31.58%)  Severe: 5 (26.32%) | OR=0.252 | 0.16 | 0.1, 0.9 | **.031** |  |
| GP usage (minutes) ^£^ | 1 | 48 | 42.52 (46.84) |  |  |  |  |  |
|  | 2 | 28 | 8.81 (9.79) | -36.077 | 12.13 | -59.9, -12.3 | **.003** | -0.881 |
|  | 3 | 20 | 70.84 (99.41) | 25.541 | 13.69 | -1.3, 52.4 | .062 | 0.380 |
| Psychological support (minutes) ^£^ | 1 | 48 | 51.96 (105.03) |  |  |  |  |  |
|  | 2 | 28 | 18.21 (50.56) | -37.516 | 17.12 | -71.1, -4 | **.028** | -0.417 |
|  | 3 | 20 | 8.25 (26.42) | -46.121 | 19.36 | -84.1, -8.2 | **.017** | -0.505 |
| A&E usage  (minutes) ^£^ | 1 | 48 | 1084.05 (3640.5) |  |  |  |  |  |
|  | 2 | 28 | 26.17 (86.86) | -1028.189 | 602.28 | -2208.6, 152.3 | .088 | -0.351 |
|  | 3 | 20 | 3.67 (9.04) | -1115.036 | 673.19 | -2434.5, 204.4 | .098 | -0.361 |
| Secondary care usage (minutes) ^£^ | 1 | 48 | 46.91 (88.89) |  |  |  |  |  |
|  | 2 | 28 | 8.93 (24.24) | -36.732 | 15.62 | -67.4, -6.1 | **.019** | -0.495 |
|  | 3 | 20 | 46.5 (62.71) | 1.335 | 17.6 | -33.2, 35.8 | .940 | 0.016 |
| Health related quality of life (EQ5D) | 1 | 48 | 0.69 (0.21) |  |  |  |  |  |
|  | 2 | 29 | 0.74 (0.17) | 0.024 | 0.02 | 0, 0.1 | .280 | 0.122 |
|  | 3 | 20 | 0.72 (0.17) | 0.036 | 0.02 | 0, 0.1 | .160 | 0.183 |
| Global Symptom Impression (PGIS) | 1 | 48 | 2.31 (0.93) |  |  |  |  |  |
|  | 2 | 29 | 2.21 (1.05) | -0.099 | 0.15 | -0.4, 0.2 | .521 | -0.102 |
|  | 3 | 20 | 2.4 (0.75) | 0.001 | 0.17 | -0.3, 0.4 | .994 | 0.001 |
| Distress (PHQ-ADS) | 1 | 48 | 23.19 (10.78) |  |  |  |  |  |
|  | 2 | 35 | 15.75 (10.96) | -5.816 | 1.18 | -8.1, -3.5 | <.001 | -0.510 |
|  | 3 | 26 | 17.96 (11.89) | -5.425 | 1.33 | -8, -2.8 | <.001 | -0.477 |

*Note*. ^£^ The mixed effects model assumptions were violated, however, once adjusted, there were minimal changes in B values, and effect sizes, so results from original models are presented.

A&E=accident and emergency, CRP= C-Reactive Protein, GP = general practitioner, PGIS= Patient Global Impression Scales of Severity, PHQ-ADS=Patient Health Questionnaire Anxiety and Depression Scale, SRDA=self-reported disease activity, SRM=standardised response mean, WBC=white blood cell count.

**Table S5**. Associations of changes in primary outcomes and distress excluding participants with recent changes to medication

|  |  | n | Correlation coefficient |  | Regression |  |  |  |
| --- | --- | --- | --- | --- | --- | --- | --- | --- |
|  |  |  | *r* |  | *B* | SE | 95%CI | *p* |
| Faecal Calprotectin | 1 | 14 | -0.352 |  | -9.219 | -15.1 | -44.2, 25.8 | .56 |
|  | 2 | 9 | - |  |  |  |  |  |
| CRP | 1 | 19 | -0.365 |  | -0.043 | -0.20 | -0.47, 0.38 | .83 |
|  | 2 | 13 | 0.393 |  | -0.012 | -0.36 | -0.84, 0.8 | .98 |
| SRDA | 1 | 25 | 0.192 |  | 0.033 | -0.03 | -0.04, 0.11 | .37 |
|  | 2 | 18 | -0.112 |  | -0.039 | -0.03 | -0.11, 0.03 | .27 |

*Note*. * indicates p<0.05 for correlation coefficients. CRP= C-Reactive Protein, SRDA= self-reported disease activity

**Table S6.** Treatment effect estimates and standardised mean difference for effectiveness adjusted for age and gender in a sensitivity analysis limiting window between study timepoint and biomarker collection at 1-month

|  | Time | n | Mean (SD) | B | SE | 95% CI | *p* | Cohen’s *d* |
| --- | --- | --- | --- | --- | --- | --- | --- | --- |
| Faecal calprotectin* | 1 | 11 | 460.82 (928) |  |  |  |  |  |
|  | 2 | 15 | 107.47 (130.02) |  |  |  |  |  |
|  | 3 | 4 | 92.5 (91.93) |  |  |  |  |  |
| CRP | 1 | 23 | 11.13 (37.02) |  |  |  |  |  |
|  | 2 | 21 | 5.29 (10.87) | 0.013 | -0.19 | (-0.4, 0.4) | .95 | 0.013 |
|  | 3 | 24 | 2.38 (3.91) | -0.007 | -0.19 | (-0.4, 0.4) | .97 | -0.007 |
| WBC | 1 | 25 | 7.27 (2.58) |  |  |  |  |  |
|  | 2 | 22 | 6.72 (2.06) | -0.415 | -0.32 | (-1, 0.2) | .19 | -0.177 |
|  | 3 | 24 | 6.63 (1.98) | -0.634 | -0.31 | (-1.3, 0) | **.05** | -0.275 |
| Ferritin* | 1 | 7 | 227.57 (499.29) |  |  |  |  |  |
|  | 2 | 6 | 127 (141.62) |  |  |  |  |  |
|  | 3 | 3 | 94.67 (136.55) |  |  |  |  |  |
| Lymphocytes | 1 | 25 | 1.84 (0.85) |  |  |  |  |  |
|  | 2 | 22 | 1.89 (0.99) | 0.13 | -0.15 | (-0.2, 0.4) | .39 | 0.143 |
|  | 3 | 25 | 2.14 (1.13) | 0.248 | -0.14 | (0, 0.5) | .09 | 0.248 |
| Monocytes | 1 | 25 | 0.49 (0.16) |  |  |  |  |  |
|  | 2 | 22 | 0.55 (0.22) | 0.045 | -0.03 | (0, 0.1) | .17 | 0.235 |
|  | 3 | 25 | 0.57 (0.2) | 0.042 | -0.03 | (0, 0.1) | .19 | 0.232 |
| Neutrophils | 1 | 25 | 4.55 (2.61) |  |  |  |  |  |
|  | 2 | 22 | 4.07 (1.48) | -0.068 | -0.07 | (-0.2, 0.1) | .34 | -0.153 |
|  | 3 | 25 | 3.9 (1.45) | -0.138 | -0.07 | (-0.3, 0) | **.05** | -0.313 |

* indicates variables with inadequate numbers to perform analysis after sensitivity criterion. CRP=C-Reactive Protein, WBC=white blood cell count.

**Table S7**. Associations with changes in primary outcomes and distress in a sensitivity analysis limiting window between study timepoint and biomarker collection at 1-month

|  |  | n | Correlation |  | Regression | SE | 95%CI | *p* |
| --- | --- | --- | --- | --- | --- | --- | --- | --- |
|  |  |  | *r* |  | B |  |  |  |
| Faecal Calprotectin^£^ | 1 |  | - |  |  |  |  |  |
|  | 2 |  | - |  |  |  |  |  |
| CRP | 1 | 16 | -0.175 |  | 1.317 | 3.92 | -7.4, 10.1 | .744 |
|  | 2 | 13 | 0.596* |  | 5.284* | 2.18 | 0.1, 10.4 | .046 |

* indicates p<0.05. ^£^ indicates variables with inadequate numbers to perform analysis after sensitivity criterion. CRP=C-Reactive Protein.
